# Supplementary material for: ANGPTL3 Variants Associate with Lower Levels of Irisin and C-Peptide in a Cohort of Arab Individuals
Source: Genes (Basel). 2021 May 17;12(5):755. doi: 10.3390/genes12050755 (PMC8170900; doi:10.3390/genes12050755)
Supplement: Supplementary file 1 [file genes-12-00755-s001.zip › genes-1198419-supplementary.pdf]

Table S1. SNP quality assessment tests for Hardy-Weinberg equilibrium (HWE).

SNP	Gene (functional consequence)	Minor/Major alleles	MAF	Genotype counts	Observed heterozygous	Expected heterozygous	*P*-value HWE
rs1748197	*DOCK7* (intronic)	A/G	0.355	40/119/121	0.425	0.458	0.241
rs12130333	*DOCK7-ATG4C* (intergenic)	T/C	0.146	10/62/209	0.220	0.249	0.057

Table S2. Clinical characteristics of the study cohort as per gender-wise distribution[@].

Traits	Male participants (Mean±SD)	Female participants (Mean±SD)	*P*-value[$] (for differences in mean values between male and female participants)
Age (in years)	46.62±12.44	45.95±12.37	0.654
Height (in meter)	1.72±0.065	1.59±0.06	$<2.2 \times 10^{-16}$
Weight (in kg)	89.07±14.62	75.33±14.86	3.48×10^{-13}
BMI (kg/m^2)	30.12±4.71	29.77±5.53	0.569
WC (in cm)	104.76±11.43	95.30±13.31	1.01×10^{-6}
HDL (in mmol/l)	1.05±0.249	1.32±0.32	6.75×10^{-13}
TC (in mmol/l)	5.25±1.19	5.52±1.24	0.0701
LDL (in mmol/l)	3.48±1.22	3.53±1.11	0.728
Non-HDL (inmmol/l)	4.19±1.21	4.13±1.16	0.654
TG (in mmol/l)	1.39±0.635	1.08±0.52	3.73×10^{-5}
FPG (in mmol/l)	6.11±1.41	5.51±1.00	2.9×10^{-4}
HbA1c (%)	6.54±1.34	6.12±1.22	0.012
Irisin (ng/ml)	564.39±179	550.92±202	0.602
IL7 (pg/ml)[@]	13.93±6.03	12.67±5.48	0.171
IL13 (pg/ml)	10.46±4.48	9.06±5.03	0.067
Insulin (pg/ml)	15.85±11.87	14.14±12.04	0.320
c-peptide (pg/ml)	2.49±1.52	2.79±1.86	0.262
ANGPTL3 (ng/ml)	36.65±9.51	37.98±10.82	0.364

TNFa (pg/ml)	125.48±32.25	129.45±32.14	0.429
Obese status	63 (obese):62 (non obese)	72 (obese):81 (non obese)	0.664
Diabetes status	60 (diabetic): 65 (non-diabetic)	60 (diabetic):93(non-diabetic)	0.177
Anti-diabetic medication	49 (med):76 (no med)	52 (no med):100	0.463
Lipid lowering medication	44(med):81	44(med):108	0.325

[@], The values for TC, and LDL were adjusted for lipid lowering medication by adopting procedures used in [Liu, D., Peloso, G., Yu, H. et al. Exome-wide association study of plasma lipids in >300,000 individuals. Nat Genet **49,** 1758–1766 (2017). https://doi.org/10.1038/ng.3977]: TC_adjusted=TC/0.8; and LDL_adjusted=LDL/0.7. The nonHDL was calculated by subtracting HDL from adjusted TC.

Table S3. Differences in the levels of c-peptide and irisin between individuals with reference homozygous genotypes and those with carrier genotypes at each of the two study variants.

**Trait**	**SNP**	**(Mean±SD) in individuals with reference homozygous genotypes (rs1748197:GG; rs12130333:CC)**	**(Mean±SD) in individuals with carrier genotypes (rs1748197:GA+AA; rs12130333:CT+TT)**	***P*-value$ (for differences in mean values between individuals having reference genotypes and carrier genotypes.**
c-peptide	rs1748197	3.155±1.819	2.25±1.55	0.0015
	rs12130333	2.91±1.79	1.94±1.27	0.0003
Irisin	rs1748197	599.45±192.83	524.9±186.7	0.027
	rs12130333	573.29±191.33	509.37±188.64	0.085

[$], Student's t-test was used for quantitative variables.

Table S4. Results of association tests for the two study variants with phenotype traits, using genetic model based on additive mode of inheritance. Association tests were adjusted for age, sex (Regular correction) and further confounders of diabetes medication (DM) and lipid lowering medication (LLM). Significant *P*-values passing the threshold for multiple testing (*P*-value ≤0.003 = 0.05/17) and significant P_{emp}-value≤0.05 are high-lighted in bold & italics font. Associations with *P*-values ≤0.05 are highlighted in bold font.

**Traits**	**SNP with effect allele**	**Correction**	**Sample size**	**β**	***P*-value[&]**	**Empirical *P*-value (P_{emp}-value)[&]**
c-peptide	rs1748197	R	160	-0.6976	***0.000127***	***0.00679***
		DM	160	-0.6944	***0.000161***	***0.00939***
		LLM	160	-0.6964	***0.000138***	***0.00739***
	rs12130333	R	161	-0.9002	***0.00032***	***0.0154***
		R+DM	161	-0.8991	***0.000335***	***0.0174***
		R+LLM	161	-0.9117	***0.000288***	***0.0167***
Irisin	rs1748197	R	217	-63.1	***0.000299***	***0.0149***
		DM	216	-67.78	***9.58E-05***	***0.0047***
		LLM	216	-63	***0.000357***	***0.0184***
	rs12130333	R	218	-72.61	***0.002135***	0.0979
		R+DM	217	-70.87	***0.002436***	0.1184
		R+LLM	217	-74.3	***0.001806***	0.0898
TG	rs1748197	R	257	-0.07759	0.1235	0.9989
		DM	256	-0.1008	**0.04564**	0.8984
		LLM	256	-0.08852	0.08188	0.9853
	rs12130333	R	258	-0.1369	**0.03926**	0.8709
		R+DM	257	-0.132	**0.04434**	0.8917
		R+LLM	257	-0.1334	**0.04469**	0.8991
FPG	rs1748197	R	239	-0.05879	0.5645	1
		DM	238	-0.1421	0.1395	0.9995

		LLM	238	-0.07696	0.4457	1
	rs12130333	R	239	-0.06309	0.6295	1
		R+DM	238	-0.09334	0.4424	1
		R+LLM	238	-0.07927	0.5372	1
HbA1c	rs1748197	R	251	0.1714	0.1094	0.9958
		DM	250	0.03895	0.6681	1
		LLM	250	0.1411	0.1676	0.9997
	rs12130333	R	252	-0.09417	0.5011	1
		R+DM	251	-0.1285	0.2672	1
		R+LLM	251	-0.09992	0.4505	1
HDL	rs1748197	R	257	0.003359	0.8989	1
		DM	256	0.01511	0.5664	1
		LLM	256	0.01106	0.6766	1
	rs12130333	R	258	0.0582	0.09481	0.9926
		R+DM	257	0.05328	0.1207	0.9986
		R+LLM	257	0.05558	0.1088	0.9969
nonHDL	rs1748197	R	256	-0.2321	0.0257	0.7363
		DM	256	-0.2417	0.0208	0.6565
		LLM	256	-0.2577	0.0113	0.4375
	rs12130333	R	257	-0.1641	0.2344	1
		R+DM	257	-0.1603	0.2461	1
		R+LLM	257	-0.1555	0.2486	1
LDL[@]	rs1748197	R	266	-0.1032	0.3132	1
		DM	265	-0.116	0.2594	1

		LLM	265	-0.129	0.1944	1
	rs12130333	R	267	7.08E-05	0.999	1
		R+DM	266	0.00088	0.994	1
		R+LLM	266	0.0087	0.946	1
TC@	rs1748197	R	270	-0.1397	0.1831	0.999
		DM	269	-0.146	0.1666	0.999
		LLM	269	-0.163	0.1134	0.999
	rs12130333	R	271	0.0145	0.9157	1
		R+DM	270	0.0156	0.9097	1
		R+LLM	270	0.0186	0.8901	1
Weight	rs1748197	R	271	-0.847	0.5094	1
		DM	270	-1.423	0.2598	1
		LLM	270	-1.269	0.3155	1
	rs12130333	R	272	-2.25	0.182	1
		R+DM	271	-2.135	0.1928	1
		R+LLM	271	-2.142	0.1932	1
BMI	rs1748197	R	275	-0.5333	0.2292	1
		DM	274	-0.771	0.07754	0.9833
		LLM	274	-0.7136	0.102	0.9963
	rs12130333	R	276	-0.9555	0.09993	0.9943
		R+DM	275	-0.9042	0.1099	0.9974
		R+LLM	275	-0.9168	0.1063	0.9974
Waist circumference	rs1748197	R	177	-0.9137	0.4845	1

		DM	177	-1.681	0.1732	0.9998
		LLM	177	-1.383	0.2642	1
	rs12130333	R	177	-2.191	0.2181	1
		R+DM	177	-2.335	0.1619	0.9996
		R+LLM	177	-2.637	0.1173	0.9986

@, The values for TC, and LDL were adjusted for lipid lowering medication by adopting procedures used in [Liu, D., Peloso, G., Yu, H. *et al.* Exome-wide association study of plasma lipids in >300,000 individuals. *Nat Genet* **49,** 1758–1766 (2017). https://doi.org/10.1038/ng.3977]: TC_adjusted=TC/0.8; and LDL_adjusted=LDL/0.7. The nonHDL was calculated by subtracting HDL from adjusted TC. [&], Significant values are indicated by bold and italics font.

Table S5. Evaluation of the association signals relating to c-peptide and irisin in the sub-cohorts of entirely diabetic individuals and of entirely non-diabetic individuals.

**Traits**	**SNP**	**Correction**	**β**	***P*-value**	**Empirical *P*-value (P_{emp}-value)**
**Sub-cohort of diabetes individuals**					
c-peptide	rs1748197	R	-0.809	0.0012	0.0732
	rs12130333	R	-0.709	0.039	0.863
irisin	rs1748197	R	-103.9	0.00011	0.0054
	rs12130333	R	-115.3	0.0022	0.106
**Sub-cohort of diabetes individuals**					
c-peptide	rs1748197	R	-0.517	0.0614	0.955
	rs12130333	R	-1.169	0.0019	0.104
irisin	rs1748197	R	-28.08	0.194	1
	rs12130333	R	-34.12	0.231	1

Table S6. Logistic regression analysis for impact of the two study variants on the disease status of the study participants.

Disease status	SNP	OR [CI]	Standard error	*P*-value
Obesity	rs1748197	0.794 [0.56-1.12]	0.176	0.191
	rs12130333	0.765 [0.48-1.21]	0.234	0.253
Diabetes	rs1748197	1.23 [0.84-1.78]	0.189	0.274
	rs12130333	1.01 [0.62-1.64]	0.248	0.967
Hypertension	rs1748197	1.004 [0.66-1.52]	0.211	0.983
	rs12130333	0.621 [0.348-1.11]	0.295	0.106

Table S7. Power Calculation for the association of the rs1748197_A variant (MAF=0.35) and rs12130333_T variant (MAF=0.15) with c-peptide and Irisin. Calculations for "gene only" hypothesis were performed using additive genetic model (AA versus AG versus GG for rs1748197; TT versus TC versus CC for rs12130333); calculations for "gene-environment GxE" hypothesis were performed using dominant genetic model (AA versus (AG+GG) for rs1748197; TT versus TC+CC for rs12130333).

Frequency	Marginal Rsq	Sample Size	Expected (±) effect size considering "Gene only" hypothesis (response variable) for c-peptide	Expected (±) effect size considering "Gene only" hypothesis (response variable) for Irisin	Expected (±) effect size considering GXE hypothesis (Irisin and E=TG)
0.15	0.001	7845	0.1083	12.039	20.40
	0.006	1304	0.2652	29.491	49.96
	0.011	710	0.3591	39.931	67.65
	0.016	487	0.4331	48.159	81.60
	0.021	370	0.4962	55.173	93.48
	0.026	298	0.5521	61.391	104.02
	0.031	249	0.6028	67.034	113.58
	0.036	214	0.6496	72.238	122.40
	0.041	187	0.6933	77.092	130.62
	0.046	167	0.7344	81.657	138.35
0.35	0.001	7845	0.0811	9.013	15.27

	0.006	1304	0.1985	22.078	37.41
	0.011	710	0.2688	29.893	50.65
	0.016	487	0.3242	36.053	61.08
	0.021	370	0.3714	41.304	69.98
	0.026	298	0.4133	45.958	77.87
	0.031	249	0.4513	50.183	85.03
	0.036	214	0.4863	54.079	91.63
	0.041	187	0.5190	57.713	97.78
	0.046	167	0.5498	61.131	103.57
